# Comparative immunological evaluation of recombinant *Salmonella* Typhimurium strains expressing model antigens as live oral vaccines

**DOI:** 10.1186/1471-2172-13-54

**Published:** 2012-09-26

**Authors:** Song-yue Zheng, Bin Yu, Ke Zhang, Min Chen, Yan-Hong Hua, Shuofeng Yuan, Rory M Watt, Bo-Jian Zheng, Kwok-Yung Yuen, Jian-Dong Huang

**Affiliations:** 1 Department of Biochemistry, the University of Hong Kong, Pokfulam, Hong Kong; 2 Department of Microbiology, the University of Hong Kong, Pokfulam, Hong Kong; 3 Oral Biosciences, Faculty of Dentistry, The University of Hong Kong, Prince Philip Dental Hospital, 34 Hospital Road, Sai Ying, Hong Kong

**Keywords:** *Salmonella* Typhimurium, Live oral vaccine, Soluble and insoluble antigens, Construction strategies, Immunological comparison

## Abstract

**Background:**

Despite the development of various systems to generate live recombinant *Salmonella* Typhimurium vaccine strains, little work has been performed to systematically evaluate and compare their relative immunogenicity. Such information would provide invaluable guidance for the future rational design of live recombinant *Salmonella* oral vaccines.

**Result:**

To compare vaccine strains encoded with different antigen delivery and expression strategies, a series of recombinant *Salmonella* Typhimurium strains were constructed that expressed either the enhanced green fluorescent protein (EGFP) or a fragment of the hemagglutinin (HA) protein from the H5N1 influenza virus, as model antigens. The antigens were expressed from the chromosome, from high or low-copy plasmids, or encoded on a eukaryotic expression plasmid. Antigens were targeted for expression in either the cytoplasm or the outer membrane. Combinations of strategies were employed to evaluate the efficacy of combined delivery/expression approaches. After investigating *in vitro* and *in vivo* antigen expression, growth and infection abilities; the immunogenicity of the constructed recombinant *Salmonella* strains was evaluated in mice. Using the soluble model antigen EGFP, our results indicated that vaccine strains with high and stable antigen expression exhibited high B cell responses, whilst eukaryotic expression or colonization with good construct stability was critical for T cell responses. For the insoluble model antigen HA, an outer membrane expression strategy induced better B cell and T cell responses than a cytoplasmic strategy. Most notably, the combination of two different expression strategies did not increase the immune response elicited.

**Conclusion:**

Through systematically evaluating and comparing the immunogenicity of the constructed recombinant *Salmonella* strains in mice, we identified their respective advantages and deleterious or synergistic effects. Different construction strategies were optimally-required for soluble *versus* insoluble forms of the protein antigens. If an antigen, such as EGFP, is soluble and expressed at high levels, a low-copy plasmid-cytoplasmic expression strategy is recommended; since it provokes the highest B cell responses and also induces good T cell responses. If a T cell response is preferred, a eukaryotic expression plasmid or a chromosome-based, cytoplasmic-expression strategy is more effective. For insoluble antigens such as HA, an outer membrane expression strategy is recommended.

## Background

Over recent years, there has been considerable interest in the use of attenuated *Salmonella* Typhimurium strains as live oral vaccines against heterologous antigens or infectious agents [1,2]. Notable advantages to their use include their ability to induce antigen-specific mucosal, humoral and cellular responses after infecting the host via mucosal routes [3-5]. *Salmonella* strains can be orally administrated, via food or drinking water, which avoids the use of injections for vaccine delivery. Furthermore, vaccine strains may be conveniently and reliably generated in large quantities by standard cell culture-based approaches, which considerably lowers the manufacturing costs [6]. Consequently, many different strategies have been utilized for the development of live recombinant *Salmonella* oral vaccines against various heterologous antigens. Some of these approaches utilize episomal expression systems, where high or low copy plasmids are used to express heterologous antigen or epitope genes under the control of various different promoter systems [7-10]. The antigenic proteins may be retained within the cell cytoplasm after expression, or may be targeted to cell surface (outer membrane) of the attenuated *Salmonella* strains. For example, the Pneumococcal surface protein A (PspA) and codon-optimized influenza hemagglutinin (HA) protein have both been expressed in the cytoplasm of attenuated *Salmonella* Typhimurium cells via high copy plasmids, for use as live oral vaccine candidates against *Streptococcus pneumonia* or the H5N1 virus [7,8], respectively. A high copy ice-nucleation protein (Inp) derived surface-expressed system was employed to produce a live recombinant *Salmonella* oral vaccine against hepatitis B virus, while a low copy OmpA-derived surface display system was used to develop a live recombinant *Salmonella* oral vaccine against HIV [9,10]. All of these vaccines were demonstrated to induce strong immune responses, especially humoral responses. In other studies, heterologous genes have been expressed in the cytoplasm of attenuated *Salmonella* via chromosome-based expression cassettes. For instance, a live recombinant *Salmonella* strain containing a chromosomally-encoded SARS-CoV nucleocapsid gene expressed sufficient cytoplasmic protein to elicit both humoral and cellular immune responses against SARS-CoV [11]. *Salmonella* has also been employed as a live delivery vector for eukaryotic expression plasmids harboring antigen genes. This strategy may avoid problems such as codon bias or low levels of antigen expression. Using this approach, live recombinant *Salmonella* was used to deliver an HA DNA vaccine against the H9N2 virus, and a VP28 DNA vaccine against the white spot syndrome virus in crayfish [12,13].

In several reports, two or three different approaches have been used to construct analogous live recombinant *Salmonella* oral vaccines that target the same pathogen, and their respective immunogenicities were subsequently compared. In studies aimed at developing a live oral *Salmonella* vaccine against *Mycoplasma hyopneumoniae*, identical Adh and NrdF antigen genes were expressed using either plasmid- or chromosome-based systems. Animal tests suggested that the vaccine strain containing the chromosome-based expression system induced a stronger humoral immune response than the one containing the plasmid-based expression system [14]. Similarly, in efforts to construct a live oral *Salmonella*-HIV vaccine, the HIV rgp120 antigen was expressed cytoplasmically using either a chromosome-based cassette, a high copy plasmid, or in the outer membrane via an OmpA expression system. Animal experiments subsequently revealed that the membrane-expressed HIV antigen was capable of inducing a better humoral response than the cytoplasmically-expressed HIV antigen expressed using the other two expression systems [10].

The enhanced green fluorescent protein (EGFP), originally isolated from the *Aequorea victoria* jellyfish, is an easily expressed, soluble and species-independent reporter protein, which emits green florescence after absorbing blue light. Its intracellular expression levels and location can be conveniently monitored by fluorescence microscopy, using a fluorescence micro-plate reader, or by fluorescence-assisted flow cytometry. Furthermore, it has been reported that it contains a mouse H2-K^d^-restricted CTL epitope, which could elicit a strong immune response [15]. All of these characteristics suggest that EGFP could be a good vaccine model antigen in mice [16].

Despite the development of numerous strategies for the construction of *Salmonella* vaccine strains, there have been relatively few studies aimed at systematically evaluating and comparing their respective immunogenic properties. Such information would provide invaluable guidance for the future rational design of live recombinant *Salmonella* oral vaccines. To directly address this issue, we construct analogous sets of live recombinant *Salmonella* Typhimurium vaccine strains expressing two model antigens: enhanced green fluorescent protein (EGFP) and the hemagglutinin epitope (residues 91–261, HAOP) from H5N1 virus (H5N1 A/Vietnam/1194/2004), via different commonly used strategies. Through systematically evaluating and comparing the immunogenicity of these strains in mice, we identified their respective advantages, as well as deleterious or synergistic effects. The construction strategies of recombinant *Salmonella* vaccine strains for various needs and different forms of antigens (soluble or insoluble antigens) were also proposed.

## Results

### Construction of recombinant *Salmonella*-EGFP strains

Six ‘single-recombinant’ SL7207-EGFP strains were first constructed to express the *EGFP* gene by a single strategy in a single chromosomal or episomal context, respectively (Table 1). Strains HP, LP and CP were each designed to express EGFP in the cytoplasm of *Salmonella* from a high copy plasmid, low copy plasmid or chromosome-based *prosseA* expression cassette, respectively. Strains HO and CO were designed to express EGFP on the outer membrane of SL7207 from a high copy plasmid, or a chromosome-based *prosseA-*lpp-ompA outer membrane expression cassette, respectively. Strain E carried a high-copy eukaryotic expression plasmid *p*EGFP driven by a eukaryotic *cmv* promoter. Consequently, EGFP would not be expected to be expressed in significant amounts until *p*EGFP was released into cells of the eukaryotic host. HP and HO sub-groups were constructed by using the high copy number plasmid with *p*UC origin as backbone. These plasmids exist in about 500–700 copies in one bacterial cell [17]. Low copy number plasmid sub-group LP was constructed using *p*15A origin in the backbone. The plasmid exists in 10–12 copies per bacterial cell [17]. Chromosome-based sub-groups CP and CO were constructed by lambda red recombineering [18] which resulted in only one copy of EGFP expressing cassette in one bacterial cell. The high-copy eukaryotic expression plasmid *p*EGFP also uses *p*UC origin as backbone. 

**Table 1:** **Plasmids and strains used in this study**

**Plasmids**	**Relevant genotype or characteristics**	**Ref. or source**
*p*Bluescript II SK	Amp^R^; Cloning vector, *p*UC origin 500–700 copy per cell	Stratagene
*p*MD18-T	Amp^R^; Cloning vector, *p*UC origin 500–700 copy per cell	Takara
*p*Sim6	Amp^R^,Lambda-red recombinase plasmid	[18]
*p*loxp-cm-loxp	Amp^R^, Cm^R^ ; *p*BSK derivative containing EGFP and loxp-cm-loxp fragment	[57]
*p*EGFP	Km^R^ ; CMV promoter drived EGFP expression, *p*UC origin 500–700 copy per cell	Lab stock
*p*ACYC177	Cm^R^ ; *p*15a origin plasmid, 10–12 copy per cell	Lab stock
*p*VAX-1	Km^R^ ; CMV promoter; *p*UC origin 500–700 copy per cell	Lab stock
*p*HP	Amp^R^; *p*BSK derivative containing *prosseA* promoter, *egfp* gene	This study
*p*HAOP	Amp^R^; *p*BSK derivative containing *prosseA* promoter, *ha(91–261)* gene	This study
*p*CP-cm	Amp^R^, Cm^R^ ; *p*BSK derivative containing *prosseA* promoter, *egfp* gene, and loxp-cm-loxp fragment	This study
*p*LP	Cm^R^ ; *p*ACYC177 derivative containing *prosseA* promoter, *egfp* gene	This study
*p*OmpA	Amp^R^; *p*MD18-T derivative containing *lac* promoter, and *lpp-ompA* gene	This study
*p*HO	Amp^R^; *p*MD18-T derivative containing *lac* promoter, and *lpp-ompA-egfp* gene	This study
*p*O-HAOP	Amp^R^; *p*MD18-T derivative containing *lac* promoter, and *lpp-ompA-ha(91–261)* gene	This study
*p*CO-cm	Amp^R^, Cm^R^ ; *p*BSK derivative containing *prosseA* promoter, *lpp-ompA-egfp* gene, and loxp-cm-loxp fragment	This study
**Strains**	**Relevant genotype or characteristics**	**Ref. or source**
***S.*typhimurium**		
SL7207	*his*G46 DEL407 [aroA::Tn10 {Tc^s^}];	B. Stocker
HP	Amp^R^; SL7207; with plasmid *p*HP	This study
LP	Cm^R^ ; SL7207; with plasmid *p*LP	This study
CP	Cm^R^ ; SL7207; *htrA<>prosseA-egfp-cm*	This study
HO	Amp^R^; SL7207; with plasmid *p*HO	This study
CO	Cm^R^ ; SL7207; *htrA<>prosseA-lpp-ompA-egfp -cm*	This study
E	Km^R^ ; SL7207; with plasmid *p*EGFP	This study
CP + HP	Cm^R^ ; Amp^R^; SL7207; CP with plasmid *p*HP	This study
CP + LP	Cm^R^ ; SL7207; CP with plasmid *p*LP	This study
CP + HO	Cm^R^ ; Amp^R^; SL7207; CP with plasmid *p*HO	This study
CP + E	Cm^R^ ; Km^R^ ; SL7207; CP with plasmid *p*EGFP	This study
E + LP	Cm^R^ ; Km^R^ ; SL7207; LP with plasmid *p*EGFP	This study
C-HAOP	Amp^R^; SL7207; with plasmid *p*HAOP	This study
O-HAOP	Amp^R^; SL7207; with plasmid *p*O-HAOP	This study
BSK	Amp^R^; SL7207; with plasmid *p*BSK	This study
ACYC177	Cm^R^ ; SL7207; with plasmid *p*ACYC177	This study
OmpA	Amp^R^; SL7207; with plasmid *p*OmpA	This study
VAX-1	Km^R^ ; SL7207; with plasmid *p*VAX-1	This study
VAX-1 + ACYC177	Cm^R^ ; Km^R^ ; SL7207; with plasmid *p*VAX-1 and *p*ACYC177	This study
***E. coli***		
DH10B	endA1 recA1 galE15 galK16 nupG rpsL ΔlacX4 Φ Φ80lacZΔM15 araD139 Δ(ara,leu)7697 mcrA Δ(mrr-hsdRMS-mcrBC) λ-	Lab stock

Five additional ‘double-recombinant’ *Salmonella*-EGFP strains were then constructed, by combining two of the above strategies (Table 1). Four of them were created from the chromosome-based EGFP expression strain, CP. This was achieved by establishing the *p*HP, *p*LP, *p*HO, or *p*EGFP plasmids within CP, generating the double-recombinant CP + HP, CP + LP, CP + HO and CP + E strains, respectively. The *p*EGFP plasmid was transformed into the LP strain to generate a further double-recombinant strain, E + LP.

### *In vitro* and *in vivo* EGFP expression of recombinant *Salmonella* strains

#### Detection of EGFP expression and cell morphology of recombinant *Salmonella*-EGFP strains

**Figure 1:**
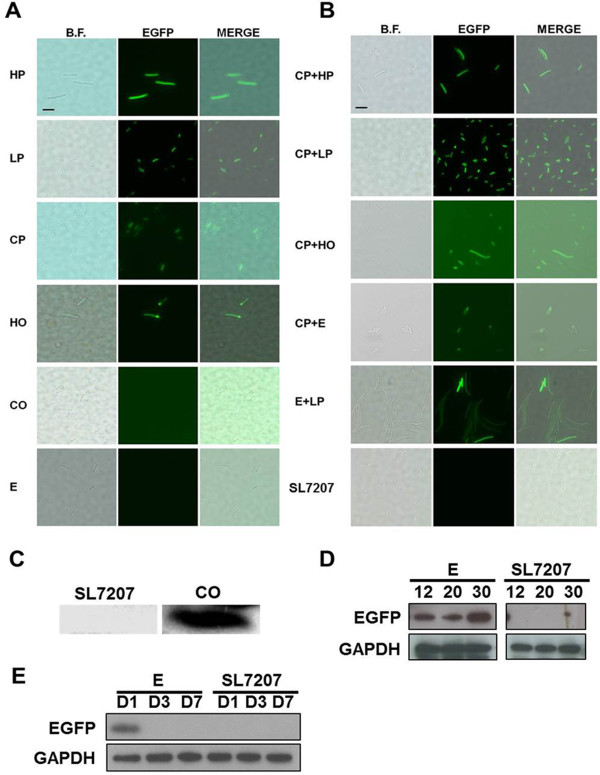
**Analysis of *in vitro *and *in vivo *expression of recombinant *Salmonella- *EGFP strains.** (**A**) and (**B**) Representative fluorescence microscopy images of EGFP antigen expression in six ‘single-recombinant’ * Salmonella * strains: HP, LP, CP, HO, CO and E (**A**) and in five ‘double-recombinant’ * Salmonella * strains: CP + HP, CP + LP, CP + HO, CP + E and E + LP (**B**). The SL7207 parent strain was used as a control. B.F. = bright-field images; MERGE = composite of bright-field and fluorescent images. Scale bar represents 5 μm. (**C**) Western blot analysis of EGFP expression in the CO strain. Expression of EGFP protein (theoretical mass ca. 39 kDa) was detected using a GFP rabbit polyclonal IgG. The SL7207 strain was used as a negative control. (**D**) Western blot analysis of EGFP expression in the Caco-2 human colon carcinoma cell line delivered by E strain. Expression of EGFP protein (theoretical mass ca. 26 kDa) was detected at 12, 20 and 30 hours after infection using a GFP mouse monoclonal IgG. The SL7207 strain was used as a negative control. The GAPDH detection was performed as the internal control. (**E**) Western blot analysis of EGFP expression in mice intestine mucosa delivered by E strain. EGFP detection was carried out on day 1, 3 and 7 after inoculation. The SL7207 strain was performed identically as a negative control. The GAPDH was detected as the internal control.

Out of the six ‘single-recombinant’ *Salmonella*-EGFP strains, green fluorescence could be directly observed in cells of the HP, LP, CP and HO strains by fluorescent microscopic analysis (Figure 1A). Since no fluorescence was observable for the CO strain, EGFP expression was confirmed by Western blotting (Figure 1C). This revealed that the EGFP gene was being translated within the CO strain. Because EGFP would not be expressed in significant amounts until *p*EGFP in strain E was released into eukaryotic cells, we detected its expression at different time points *in vitro* and *in vivo*, respectively, using Western blotting. *In vitro*, the EGFP expression was detected by infecting the Caco-2 human colon carcinoma cell line with E strain. Figure 1D showed that EGFP could be successfully delivered into eukaryotic cells by E strain *in vitro*, and its expression was found within the eukaryotic cells at 12, 20 and 30 hours after infection. Furthermore, the EGFP expression delivered by E strain was also investigated in mice intestine on day 1, 3 and 7 after inoculation of 1 × 10^11^ bacterial cells. Western blotting result (Figure 1E) showed that EGFP expression was observed in mice intestinal mucosa on day 1 after inoculation.

**Figure 2:**
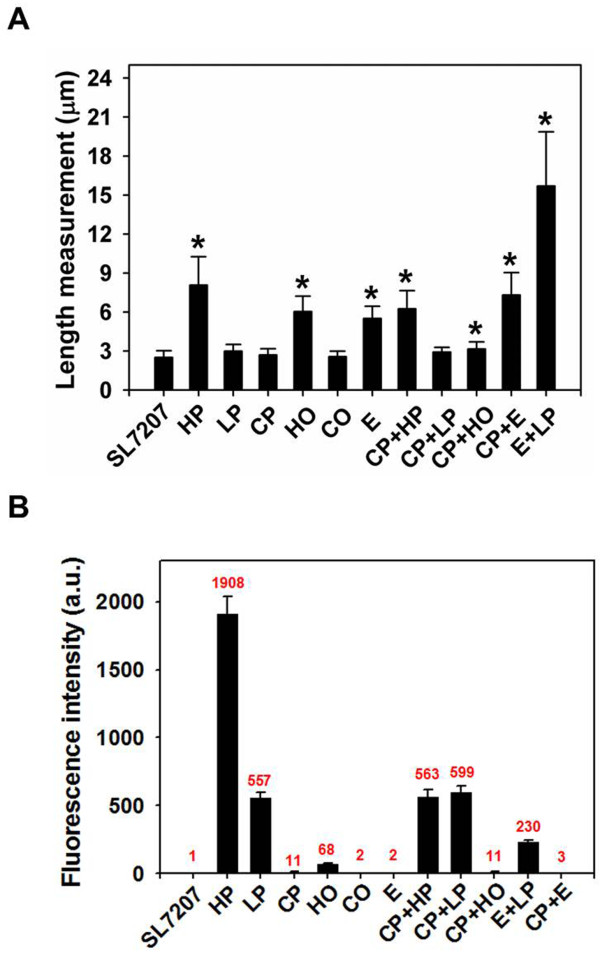
**Cell length measurement anddetermination of EGFP expression levels for recombinant *Salmonella *strains.** (**A**) Cell length measurements for the eleven recombinant *Salmonella* strains. Each bar in the graph represents the mean length of 20 bacterial cells randomly selected from multiple microscopic fields for each strain, as measured using Image J software. Error bars show the standard deviation. An asterisk indicates that the mean length of the cells were significantly different from those of the SL7207 parent strain, as determined by Student *T*-test (*P* < 0.05). (**B**) Intensity of EGFP fluorescence emitted by each of the eleven recombinant *Salmonella* strains. SL7207 was included as a negative control. Measurements correspond to the average fluorescence emitted by 1 × 10^8^ freshly-cultured bacterial cells (in 96-well micro-titer plates; excitation 488 nm, emission 518 nm); time resolved fluorescence of 500 ms. Bars in the graph represent the average fluorescence intensities from three independent experiments, ± standard deviation.

Filamentation of bacterial cell (the elongated, rod-like cell morphology) was clearly observable for the HP, HO and E strains. Consequently, the average cell lengths for each of the created strains were determined using the Image J software package, and were compared with those of the parent SL7207 strain. It is shown in Figure 2A that the average lengths of the HP, HO and E strains were 8.1 μm, 6.0 μm and 5.5 μm, respectively, which was considerably longer than the average length of the SL7207 cells, which was 2.5 μm [19]. 

Levels of EGFP-derived green fluorescence in the five double-recombinant strains were similarly analyzed by fluorescence microscopy (Figure 1B). Fluorescence could be clearly observed for cells of each of the five strains. With the exception of the CP + LP strain, cells were highly elongated compared with the SL7207 strain. Analysis of average cell length using Image J software (Figure 2A) indicated that cell filamentation of E + LP strain were the most highly elongated; with an average length of 15.7 μm. CP + HP, CP + HO and CP + E cells were also significantly filamented, with average lengths of 6.2 μm, 3.2 μm and 7.3 μm, respectively.

#### EGFP expression levels in recombinant *Salmonella* strains

To quantify EGFP expression levels within the eleven recombinant *Salmonella*-EGFP strains, the intensity of green fluorescence for 1 × 10^8^ freshly cultured planktonic cells were determined using a micro-plate reader (Figure 2B). For the three ‘cytoplasmic expression’ single-recombinant strains (i.e. HP, LP, and CP), there were clear differences in fluorescence levels. The HP strain exhibited the highest fluorescence intensity levels (1.9 × 10^3^ a.u.), followed by LP (5.6 × 10^2^ a.u.), with CP being barely fluorescent (11 a.u.). For the two membrane-based expression strains, fluorescence levels were only significant for the HO strain (68 a.u.), with the CO strain (2 a.u.) being essentially non-fluorescent; practically the same as the SL7207 strain (1 a.u.). Fluorescence levels for the E strain, which carries the eukaryotic EGFP-expression plasmid, were similarly negligible (2 a.u.). These results clearly indicated that the fluorescence intensities observed within freshly cultured, planktonic cells were highest for the cytoplasmic-expression strains, and were also positively correlated to the plasmid copy numbers. The data also match the phenotypes of the strains, as observed using fluorescence microscopy (Figure 1A). In summary, the EGFP-expression levels in the six ‘single-recombinant’ *Salmonella* strains were in the order (highest to lowest): HP > LP > HO > CP > CO and E (p < 0.01).

The intensities of EGFP fluorescence in the five ‘double-recombinant’ strains were not enhanced, as had initially been expected (Figure 2B). In contrast, for four of the five double-recombinant strains, green fluorescence intensities were significantly lower than in the respective single-recombinant parent strains. The fluorescence intensity of the CP + HP strain (5.6 × 10^2^ a.u.) was more than three-fold lower than the parent HP strain (1.9 × 10^3^ a.u., p < 0.01). The fluorescence of the CP + HO strain (11 a.u.) was essentially identical to the CP parent (11 a.u.), and more than 6-fold lower than the HO strain (68 a.u., p < 0.01). The EGFP expression levels for the E + LP strain (2.3 × 10^2^ a.u.) were less than half those determined for the LP strain (5.6 × 10^2^ a.u., p < 0.01), and the fluorescence of the CP + E strain (3 a.u.) were almost 4-fold lower than those of the CP strain (p < 0.01). Of these five combinations, only CP + LP (6.0 × 10^2^ a.u.) maintained EGFP fluorescence levels that were similar to those of the parental strain, LP (5.6 × 10^2^ a.u.). This indicated that EGFP expression levels within the double-recombinant *Salmonella* strains were generally greatly reduced, when compared to their parental strains; being listed in the following order: CP + HP, CP + LP > E + LP > CP + HO > CP + E (p < 0.01).

#### *In vitro* growth rates of recombinant *Salmonella*-EGFP strains

In order to investigate whether single and double recombinant strategies would affect the growth of recombinant strains which may further influence bacterial infection abilities *in vivo*, the doubling times of the six single and five double-recombinant *Salmonella*-EGFP strains were measured in liquid LB medium supplemented with antibiotics *in vitro*.

Table 2 shows the growth rates of the six single-recombinant *Salmonella*-EGFP strains. The doubling times of the HP, LP, CP, HO, CO and E strains were 33.2 ± 4.0, 39.2 ± 1.5, 44.2 ± 2.6, 38.9 ± 3.0, 52.5 ± 2.6 and 33.0 ± 3.0 minutes, respectively. Their doubling times were all significantly higher than that of the parental strain SL7207 (26.3 ± 0.9 min, p < 0.05). Comparing the six recombinant strains, the growth rate of the chromosome-based outer-membrane strain CO was considerably slower than that of the other five strains (p < 0.01). Furthermore, the growth rates of strains containing high or low copy-EGFP plasmids were also compared with those of strains containing the corresponding empty vectors. Strains HP and E exhibited similar growth rates to the strains containing the corresponding empty vectors BSK (35.0 ± 1.4 min) and VAX-1 (31.2 ± 1.9 min). This indicated that the metabolic burden associated with maintaining the *p*BSK and *p*VAX-1 high copy plasmids was primarily responsible for the increase in doubling times for the HP and E strains. The growth rates of the LP and HO strains were considerably different to those of the corresponding empty-vector strains ACYC177 (42.8 ± 1.7 min, p < 0.01) and OmpA (35.8 ± 1.2 min, p < 0.05). This suggested that both EGFP expression and plasmid burden affected their growth rates.

**Table 2:** ***In vitro *doubling times of six single-recombinant *Salmonella *-EGFP strains **

** Strains**	SL7207*	**HP**	**BSK**	**LP**^**(a)**^	**ACYC177**	**CP**	**HO**^**(b)**^	**OmpA**	CO**	**E**	**VAX-1**
DT (min)	26.3 ± 0.9	33.2 ± 4.0	35.0 ± 1.4	39.2 ± 1.5	42.8 ± 1.7	44.2 ± 2.6	38.9 ± 3.0	35.8 ± 1.2	52.5 ± 2.6	33.0 ± 3.0	31.2 ± 1.9

* The doubling time of the SL7207 strain was significantly different from those of the six single-recombinant strains (p < 0.05); ** The doubling time of the CO strain was significantly increased compared to those of the other five single-recombinant strains (p < 0.01); (a) A significant difference was observed between the doubling times of the LP strain and the strain containing the corresponding empty vector ACYC177 (p < 0.01); (b) The doubling times of the HO and OmpA strains are significantly different (p < 0.05).

The growth rates of five double-recombinant *Salmonella*-EGFP strains were also measured, and are described in Table 3. The doubling times of strains CP + HP, CP + LP, CP + HO, CP + E, E + LP were: 58.3 ± 2.5, 51.0 ± 2.3, 105.9 ± 7.4, 65.7 ± 2.5 and 68.3 ± 9.9 minutes, respectively. These values were considerably higher than the doubling time of the SL7207 strain (p < 0.01). Also, we found that their growth rates were significantly slower than those of the corresponding single-recombinant parental strains (p < 0.01 or p < 0.05; described in Table 3). The growth rate of strain VAX-1 + ACYC177, which contains two empty vectors: *p*VAX-1 and *p*EGFP, was measured as a control for the E + LP strain. Experiments revealed that there were no significant differences in the growth rates of these two strains. Taken together, these results indicated that double-recombinant strategies seriously reduced the growth rates of these five strains comparing to that of strains with single strategies and SL7207.

**Table 3:** ***In vitro *doubling times of five double-recombinant *Salmonella *-EGFP strains**

** Strains**	SL7207**	**CP + HP**^**(1)**^	**CP + LP**^**(2)**^	**CP + HO**^**(1)**^	**CP + E**^**(1)**^	**E + LP**^**(1)**^	**VAX-1 + ACYC177**
DT (min)	26.3 ± 0.9	58.3 ± 2.5	51.0 ± 2.3	105.9 ± 7.4	65.7 ± 2.5	68.3 ± 9.9	56.7 ± 6.6

** the doubling time of the SL7207 strain was significantly different from those of five double-recombinant strains (p < 0.01); Strains marked with (1) or (2) indicates their doubling times were significantly increased compared to their respective single-recombinant parental strains. (1) p < 0.01; (2) p < 0.05.

#### *In vitro* stability of EGFP expression constructs under antibiotic pressure

To maximize plasmid stability and copy-number within the recombinant strains, two strategies were used. Firstly, antibiotics were supplied throughout the entire experimental process. Secondly, all strains were freshly cultured for 10 hours, diluted 1:10 with fresh medium, and then cultured until the OD_600_ reached 2 a.u. Immediately prior to oral administration, the stability of the EGFP expression constructs within each of the eleven *Salmonella*-EGFP strains were evaluated using colony forming unit (CFU) assays.

Table 4 summarizes the results of these assays, which evaluate the retention of functional antibiotic-resistance gene cassettes within the six single-recombinant *Salmonella*-EGFP strains. Three plasmids (*p*LP, *p*HO, *p*EGFP) and two chromosome-based expression cassettes (CP and CO strains) exhibited good stability, with 100.0% survival rate on the selection plates. However, for the HP strain, only 88.9% of CFUs maintained *p*HP. This indicated that EGFP expression in the HP strain may be less stable than the other strains, thereby possibly affecting its immunogenic potency.

**Table 4:** **Stability of antibiotic markers in six single-recombinant *Salmonella *-EGFP strains**

**Strain**	** Origin**	**Detection rate for antibioticmarker (%)**
** HP**	*p*UC	88.9
** LP**	*p*15A	100.0
** CP**	Chromsome-based	100.0
** HO**	*p*UC	100.0
** CO**	Chromsome-based	100.0
** E**	*p*UC	100.0

Since the reduction in EGFP expression levels for the five double-recombinant strains may be related to the relative stability of the two EGFP expression cassettes, their respective stabilities were also evaluated using CFU assays, performed immediately prior to oral administration. It may be seen in Table 5 that both expression cassettes were only completely stable (i.e. detected in 100.0% of CFUs) in two of the five double-recombinant strains: CP + LP and CP + E. The chromosome-based EGFP expression cassettes (from the parent CP strain) as well as the low-copy plasmids (*p*15A-replicons, in the LP strains) appeared to be very stable, with a 100.0% detection rate. However, the high copy plasmids (*p*UC-replicons) in the double-recombinant strains were significantly less stable. The detection rates for *p*HP in the CP + HP strain, *p*HO in the CP + HO strain, and *p*EGFP in the E + LP strain were 0.8%, 60.0% and 50.0%, respectively. Even though the *p*15A and *p*UC-based plasmids should be entirely compatible within the double-recombinant E + LP strain (and maintained using orthogonal antibiotic resistance strategies), the high-copy plasmid exhibited poorer stability. It may be noted that the relative differences in fluorescence levels between the single and double-recombinant strains (Figure 2B) correlates-well with the plasmid stability properties revealed by the CFU assays (Table 5).

**Table 5:** **Stability of antibiotic markers in the five double-recombinant *Salmonella *-EGFP strains**

** Strains**	**Origin 1**	**Detection rate for antibioticmarker 1 (%)**	**Origin 2**	**Detection rate for antibioticmarker 2 (%)**	**Combined detection rate forantibiotic markers (%)**
**CP + HP**	Chromosome-based	100.0	*p*UC	0.8	0.8
**CP + LP**	Chromosome-based	100.0	*p*15A	100	100.0
**CP + HO**	Chromosome-based	100.0	*p*UC	60.0	60.0
**CP + E**	Chromosome-based	100.0	*p*UC	100.0	100.0
**E + LP**	*pUC*	50.0	*p*15A	100.0	50.0

#### *In vivo* survival and infection ability of six single-recombinant *Salmonella* strains and stability of EGFP expression constructs

To evaluate the survival and infection abilities of the six single-recombinant *Salmonella*-EGFP strains and the stabilities of the EGFP expression constructs *in vivo*; fecal samples, as well as spleen and mesenteric lymph node samples were collected from each group of mice on days 1, 3 and 7 after the oral administration of 1 × 10^11^ bacterial cells, respectively. CFU assays were carried out using LB agar plates with or without the appropriate antibiotics. The SL7207 strain was treated identically as a control.

**Figure 3:**
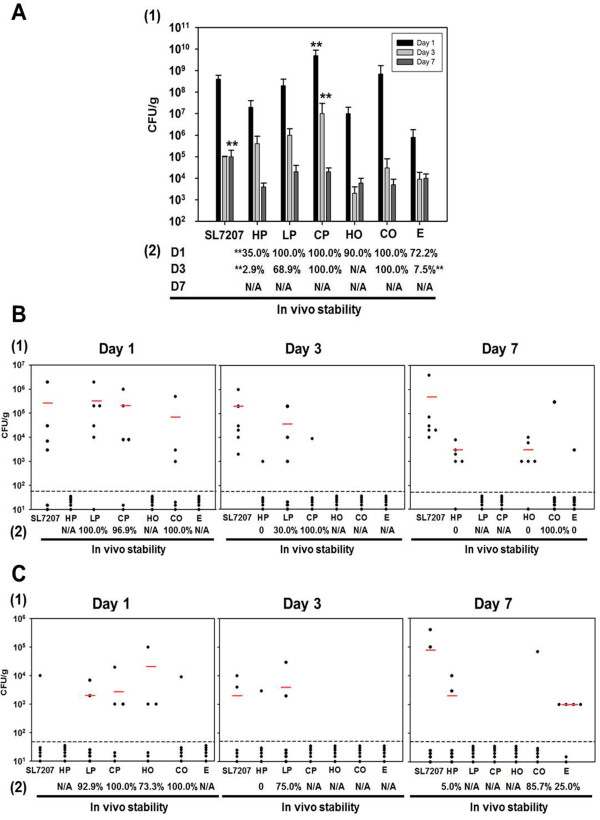
***In vivo *survival, infection ability and construct stability of six single-recombinant *Salmonella *-EGFP strains.** (**A**) Survival ability and construct stability of the six single-recombinant * Salmonella *-EGFP strains in mice feces. A (1) CFU results for the six single-recombinant strains in mice feces, on days 1, 3 and 7 after oral inoculation. Each bar in the graph represents the mean CFU/g of mice fecal samples randomly collected from six mice in each group. Error bars show the standard deviation. ** indicates that the mean CFU/g was significantly different from those of other strains (* P * < 0.01). The SL7207 strain was treated identically as a control. A (2) Construct retention rates for the six single-recombinant strains in mice feces on days 1, 3 and 7 after inoculation. ** indicates that the mean of the construct retention rate in this group was significantly different from those of the other strains (* P * < 0.01). (**B**) and (**C**) show the infection ability and construct stability results for the six single-recombinant * Salmonella *-EGFP strains in mice spleen (**B**) and mesenteric lymph nodes (**C**). B (1) and C (1) show the CFU results in mice spleen and mesenteric lymph nodes on days 1, 3 and 7 after inoculation, respectively. Each ● represents one mouse. Red lines indicate the average CFU/g for each strain. The SL7207 strain was used as a control. B (2) and C (2) show the construct retention rates for the six single-recombinant strains in mice spleen and mesenteric lymph nodes on days 1, 3 and 7 after inoculation, respectively. In A (2), B (2) and C (2), each percentage is the mean construct detection rate based on six mice samples in each group. N/A represents the construct detection rate could not be accurately calculated at this time point.

Enumeration of CFUs in feces [Figure 3A (1)] showed that 10^5^-10^9^ cfu/g of the *Salmonella* cells were present on day one, for the seven administration groups. The CP strain was present in the highest levels in the feces, with 10^9^ cfu/g (p < 0.01). The stability assays [Figure 3A (2)] suggested that the chromosome-based expression strains (CP and CO), as well as the strain with the low copy plasmid (LP), exhibited good stabilities, with 100.0% survival rates on the selection plates. However, strains containing the high copy prokaryotic or eukaryotic plasmids: HP, HO and E, appeared to be less stable; with detection rates of 35.0% (p < 0.01), 90% and 72.2%, respectively. On day 3, fecal bacterial cells in the seven administration groups declined to 10^3^-10^7^ cfu/g. The CP strain still exhibited the best survival ability, with 10^7^ cfu/g (p < 0.01). Regarding the stability of the constructs, the CP and CO strains remain good stability, with 100% detection rates on the antibiotic selection plates. But the low copy plasmid within the LP strain was somewhat less stable, declining from 100% to 68.9%. Furthermore, there was a considerable loss of the high copy plasmids within the HP, HO and E strains. Only 2.9% and 7.5% of CFUs corresponding to the HP and E strains contained high copy plasmids, respectively, whilst the *p*HO plasmid in the HO strain declined to very low levels that could not be accurately calculated. The HP, HO and E recombinant strains were significantly less stable than the CP, CO and LP strains (p < 0.01). On day 7, fecal bacterial cells from the six recombinant groups decreased to very low levels, of around 10^3^-10^4^ cfu/g, whilst the SL7207 group showed the best reserve, at around 10^5^ cfu/g (p < 0.01). Since construct stability could not be accurately calculated at these low levels, this data is not shown here. In summary, the CP chromosome-based expression strain exhibited the highest survival abilities in feces on day 1 and 3 after inoculation. The high copy prokaryotic or eukaryotic plasmids in the HP, HO and E strains appeared to be less stable in fecal samples than the chromosome-based expression cassettes in the CP and CO strains, or the low copy plasmid in the LP strain.

The infection abilities of six single-recombinant *Salmonella*-EGFP strains were determined by detecting their presence within the mouse spleens and mesenteric lymph nodes, using the SL7207 strain as a control. Figure 3B (1) & (2) shows the spleen infection results for the recombinant strains. On day 1, spleen infection was observed in the SL7207, LP, CP and CO groups. For the SL7207 group, *Salmonella* was found in 66.7% of mice spleens (4 out of 6 mice) with an average of 4 × 10^5^ cfu/g. 83.3% of mice (5 out of 6 mice) in the LP group were infected with *Salmonella*, with an average of 4 × 10^5^ cfu/g. The detection rate for the low copy plasmid was 100.0%. For the CP and CO groups, the spleen infection rates were 66.7% (4 out of 6 mice) and 50.0% (3 out of 6 mice); with an average of 2 × 10^5^ cfu/g and 8 × 10^4^ cfu/g, respectively. Their chromosome-based expression cassettes showed good stabilities, with detection rates of 96.9% and 100.0%, respectively. However, no *Salmonella* infection could be detected for the HP, HO and E strains, which contained the high copy prokaryotic or eukaryotic plasmids. On day 3, the spleen infection levels for the recombinant strains had significantly declined; however, the spleen infection rate in the SL7207 group increased to 100.0% (6 out of 6 mice) with an average of 2 × 10^5^ cfu/g. Only 16.7% of mice (1 out of 6 mice) in the HP and CP groups had *Salmonella* infections in their spleen; with 1 × 10^3^ cfu/g and 9 × 10^3^ cfu/g, respectively. The construct detection rate in HP group was 0, while that in the CP group was 100.0%. For the LP group, the spleen infection rate declined to 50% (3 out of 6 mice) with an average of 4 × 10^4^ cfu/g. The low copy plasmid stability also declined to 30.0%. Even so, the spleen infection rate for the LP group was still higher than those for the HO, CO and E groups; for which no infection was observed. On day 7, the spleen infection rate in the SL7207 administration group was still 100.0%, with an average of 7 × 10^5^ cfu/g. For the HP and HO groups, 83.3% of mice spleens (5 out of 6 mice) tested positive for *Salmonella* infection, with an average 3 × 10^3^ of cfu/g. However, no high copy plasmids could be detected in cells of either of these two strains. For the CO and E strains, only 16.7% of mice (1 out of 6 mice) had spleens infected with *Salmonella* cells, with 3 × 10^5^ cfu/g and 3 × 10^3^ cfu/g, respectively. The stability of the chromosome-based expression cassette within the CO strain remained at 100.0%, but no *p*EGFP plasmid could be isolated in the E group. Taken together, the results showed that all six of the recombinant *Salmonella*-EGFP strains could infect the spleen, but at different time points. The spleen infection rates for the recombinant strains were noticeably lower than those for the SL7207 group on day 3 and day 7. On day one, the LP low copy plasmid strain, and the CP and CO strains containing the chromosome-based expression cassettes had higher spleen infection rates with good stability, compared to the HP, HO and E strains containing high copy prokaryotic or eukaryotic plasmids. Even though infection was observed, the strains containing high copy prokaryotic or eukaryotic plasmid had noticeably poorer stability on day 7.

The mesenteric lymph node infection results for the recombinant strains are shown in Figure 3C (1) & (2). On day 1, *Salmonella* infections were observed in the SL7207, LP, CP, HO and CO groups; while no infection was detected in the HP and E groups. Only 16.7% mice (1 out of 6 mice) in the SL7207 and CO groups had *Salmonella* infection in their lymph nodes, with levels of 1 × 10^4^ cfu/g and 9 × 10^3^ cfu/g, respectively. The detection rate for the chromosome-based expression cassette within the CO strain was 100.0%. In the LP group, the lymph node infection rate was 33.3% (2 out of 6 mice), with an average of 2 × 10^3^ cfu/g. For the CP and HO groups, 50.0% of the lymph nodes (3 out of 6 mice) tested positive for *Salmonella* infection, with an average of 4 × 10^3^ cfu/g and 2 × 10^4^ cfu/g, respectively. The stability of the EGFP expression cassettes in the LP, CP and HO groups were 92.9%, 100.0% and 73.3%, respectively. On day 3, 33.3% of mice (2 out of 6) in the SL7207 and LP groups had lymph node infections, with an average of 2 × 10^3^ cfu/g and 5 × 10^3^ cfu/g, respectively. The detection rate for low copy plasmids in the LP strain was 75%. In the HP group, only 16.7% of mice (1 out of 6 mice) tested positive for *Salmonella* infection, with an average of 3 × 10^3^ cfu/g; however, no *p*HP plasmid could be isolated. For the other four groups (CP, HO, CO and E), no *Salmonella* infection was detected in the lymph nodes. On day 7, the lymph node infection rates in the SL7207 and HP groups were 33.3% (2 out of 6 mice) with an average of 8 × 10^4^ cfu/g and 2 × 10^3^ cfu/g, respectively. The detection rate for the high copy plasmid *p*HP was only 5.0%. For the CO group, 16.7% mice (1 out of 6 mice) tested positive for *Salmonella* infection in the lymph nodes, with average levels of 7 × 10^4^ cfu/g. The stability of the chromosome-based expression cassette within the CO strain was 85.7%. Furthermore, the infection rate for the E group increased to 66.7%, with average counts of 1 × 10^3^ cfu/g. However, the detection rate for the high copy plasmid *p*EGFP was only 25.0%. Considering these results as a whole, all the recombinant strains were able to infect the mice mesenteric lymph nodes at different time points; however, very few high copy plasmids could be detected in the plasmid-based recombinant strains on day 7.

### *In vivo* immune response to recombinant *Salmonella*-EGFP strains

#### EGFP-specific IgG responses in infected mouse sera

**Figure 4:**
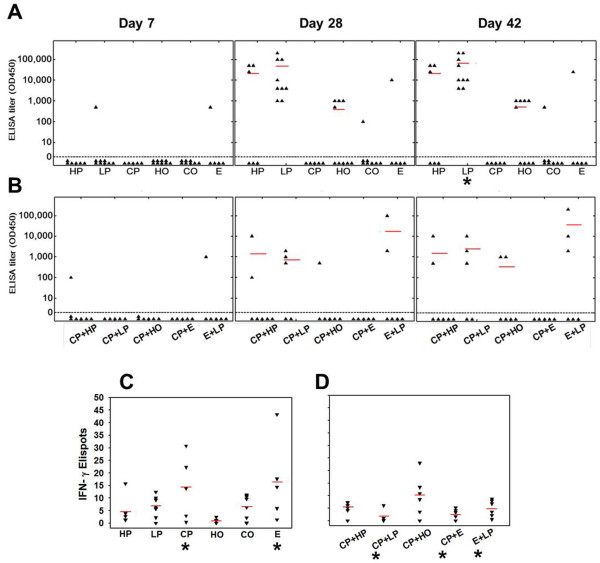
*** In vivo *immune response against recombinant *Salmonella *-EGFP strains. ** The eleven recombinant * Salmonella *-EGFP strains were respectively administrated to one of eleven groups of mice (5–10 mice for each group), with an additional group of non-infected mice kept as a negative control. For each group, 1 × 10^11^ bacteria were used to prime each mouse for 3 days, and boost on day 21 and 35, by oral gavage. (**A**) and (**B**) summarize the ELISA results showing the EGFP-specific IgG responses raised by the six single-recombinant * Salmonella * strains (**A**) and the five double-recombinant * Salmonella * strains (**B**). ELISA assays were performed using mice sera collected on day 7, 28 and 42. * underneath the LP strain in (**A**) indicates the mean ELISA titer raised by LP strain is significantly higher than those of other strain, as determined by One-Way ANOVA (* P * < 0.05). (**C**) and (**D**) summarize the mice T cell anti-EGFP IFN-γ responses raised by the six single-recombinant *Salmonella * strains (**C**), and the five double-recombinant *Salmonella* strains (**D**), using mice splenocytes collected on day 42. The ratio of spot numbers counted for the immunized mice, to those counted for non-infected mice, were used to evaluate the anti-EGFP IFN-γ response. * underneath the CP and E strain in (**C**) indicates the mean ELISpot ratios raised by CP and E strain were significantly higher than those of strains with high copy plasmids HP and HO; * underneath the CP + LP, CP + E and E + LP strains in (**D**) indicates the mean ELISpot ratios raised by these strains were significantly lower than their relative single parental strains, as determined by One-Way ANOVA (* P * < 0.05). Each ▴ represents one mouse. In (**A**) and (**B**), red lines indicate the average titers for each strain. In (**C**) and (**D**), red lines indicate the average ratios for each strain.

In order to compare the humoral immune responses mounted against eleven recombinant *Salmonella*-EGFP strains, ELISA assays were performed to test the anti-EGFP IgG responses using blood sera of infected mice on the 7^th^, 28^th^ and 42^th^ day after immunization. Results (Figure 4A) indicated that anti-EGFP IgG responses of mice immunized with six single-recombinant *Salmonella*-EGFP strains were very weak on the 7^th^ day after immunization. Only two mice, from the LP and E groups respectively, had a 1:500 anti-EGFP IgG titer. After receiving boosts on day 21 and 35, the anti-EGFP IgG responses in the mice were greatly increased. In the cytoplasmic expression group (HP, LP and CP), 50.0% of mice (3 of the 6 mice) in the HP subgroup had elevated anti-EGFP IgG responses. The average titers were around 1:21,000 on days 28 and 42. 100.0% of mice (all 9 mice) in the LP subgroup had strong anti-EGFP IgG responses, and the highest titer reached 1:200,000. The average titers were 1:47,000 on day 28, and 1:65,000 on day 42. Notably, no response could be detected in the CP subgroup even after two boosts. In the membrane-based expression group, the response rate for anti-EGFP IgG in the HO subgroup was 44.4% (4 out of 9 mice) with an average titer of 1:390 on day 28, and 55.6% (5 out of 9 mice) with an average titer 1:500 on day 42. Only 12.5% of mice (1 of 8 mice) in the CO subgroup had an anti-EGFP IgG response, with a titer of 1:100 on day 28, and 1:500 on day 42. In the eukaryotic expression plasmid group, only 20.0% of mice (1 of 5 mice) responded, having a 1:10,000 anti-EGFP IgG titer on day 28, and a 1: 25,000 titer on day 42. Collectively, based on the response rates, anti-EGFP IgG response of strains containing either high or low-copy prokaryotic expression plasmids were much better than those containing a chromosome-based expression cassette or an eukaryotic expression plasmid; especially the LP strain, which had significantly higher average anti-EGFP IgG titers on day 42 (p < 0.05).

Humoral immune response assays were also performed for the five double-recombinant *Salmonella*-EGFP strains, to investigate whether these two-gene constructs were capable of inducing stronger B cell responses. The results from analogous sets of ELISA experiments are shown in Figure 4B. Anti-EGFP IgG responses in all five mice groups were very weak on 7^th^ day after immunization. Only 14.3% of mice (1 of 7 mice) in the CP + HP group and 16.7% of mice (1 out of 6 mice) in the E + LP group generated a humoral response, with titres of 1:100 and 1:1,000, respectively. After two boosts on day 21 and 35, the response rate for anti-EGFP IgG in each group had increased. 28.6% of mice (2 of 7 mice) in the CP + HP group had anti-EGFP IgG responses, with average titers of 1:1,400 on day 28, and 1:1,500 on day 42. In the CP + LP group, 60.0% of mice (3 out of 5 mice) had anti-EGFP IgG responses, with average titers of 1:700 on day 28, and 1:2,500 on day 42. For the CP + HO strain, only 16.7% of mice (1 mouse out of 6) had developed a response by day 28; and by day 42, 33.3% (2 of 6 mice) had mounted anti-EGFP IgG responses, with average titers of ca. 1:330. In the E + LP group, 33.3% of mice (2 out of 6 mice) developed anti-EGFP IgG responses, with average titers of 1:17,000 on day 28; and 50.0% of mice (3 out of 6 mice) generated anti-EGFP IgG responses, with average titers of 1:35,000 on day 42. Notably, no response could be detected in the CP + E group even after two boosts. In brief summary, up to day 42, the humoral response rates against the five double-recombinant *Salmonella*-EGFP strains did not increase as was initially expected. They were lower than the humoral response rates observed for the respective single-recombinant parental strains.

### Interferon-gamma (IFN-γ) responses to recombinant *Salmonella*-EGFP strains

The T cell anti-EGFP IFN-γ responses against the eleven recombinant *Salmonella*-EGFP strains were also determined within immunized mice. An equally sized group of uninfected mice was included as a negative control. The anti-EGFP IFN-γ responses were evaluated by determining the ratio of ELISpot numbers counted for the respective groups of immunized mice, divided by the numbers counted for the control mice.

Figure 4C summarizes the ELISpot results obtained for the six single-recombinant *Salmonella*-EGFP strains. For the cytoplasmic expression group, the average ratios for the HP, LP and CP subgroups were 4.40, 6.51 and 14.05, respectively. For the membrane expression group (HO and CO), the CO subgroup had an average ratio of 6.39, whilst a ratio of 0.92 was obtained for the HO subgroup. The eukaryotic expression plasmid strain (E) generated anti-EGFP IFN-γ responses with an average ratio of 16.55. Comparing the average ratios obtained for the six recombinant strains, the strains containing the eukaryotic expression plasmid (E) or the chromosome-based expression cassette (CP) were capable of inducing stronger anti-EGFP IFN-γ responses than strains containing high-copy prokaryotic plasmids (HP and HO) (p < 0.05). Interestingly, this general trend is almost the opposite of that noted for the humoral immune responses.

The anti-EGFP IFN-γ responses raised by the five double-recombinant *Salmonella*-EGFP strains were also evaluated, to investigate whether strains containing two EGFP expression cassettes induced stronger T cell responses than the corresponding single-recombinant strains. As shown in Figure 4D, when CP was combined with HP, LP, HO, or E, the average ratios of anti-EGFP IFN-γ responses in mice were 5.33, 1.61, 9.90 and 2.87, respectively. The average ratio of anti-EGFP IFN-γ responses for the E + LP immunized mice *versus* the control set was 4.95. In summary, the T cell responses against the five double-recombinant *Salmonella*-EGFP strains had not been enhanced as was initially expected. Responses against several strains, such as CP + LP, CP + E and E + LP, were significantly lower than the respective single-recombinant parental strains CP and E (p < 0.05).

### Construction and expression analysis of two recombinant *Salmonella*-HA strains

In the first set of experiments, EGFP was used as a model antigen to study and compare the immunogenic potency of various recombinant *Salmonella* strains, since previous studies have shown this protein to be highly expressed in a soluble form within *Salmonella*[20-22]. However, many other heterologous antigens, such as viral proteins, may not be readily expressed in *Salmonella* in a soluble form, due to factors such as codon bias, amino acid composition or protein stability. Therefore, we chose the HA epitope (91aa-261aa) from the avian influenza H5N1 virus as a second model antigen. Considering the codon bias between *Salmonella* and influenza virus, the gene sequence encoding the HA fragment (HAOP) was first codon optimized for expression within *Salmonella*, and prepared synthetically by a commercial supplier (GenScript). The HAOP fragment was subcloned into sets of plasmids analogous to those used for the EGFP gene, in order to construct analogous expression systems within the parental *Salmonella* SL7207 strain.

**Figure 5:**
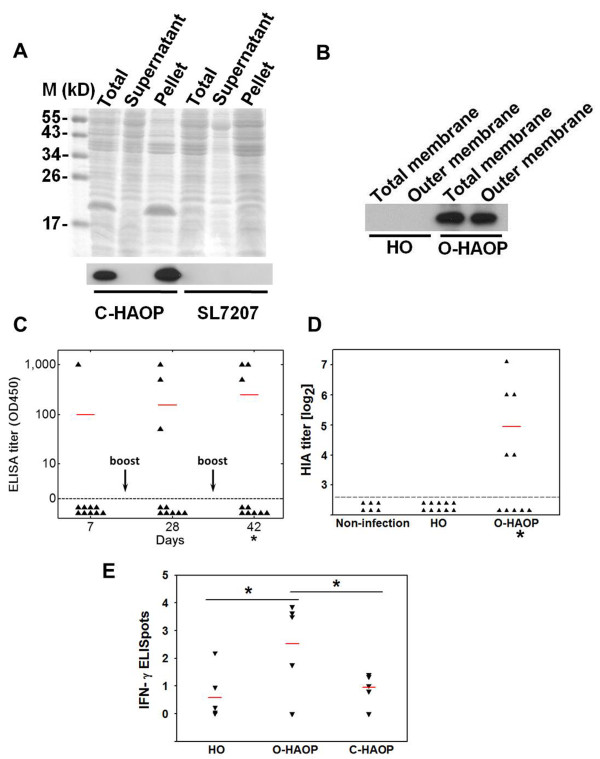
**Analysis of HA-antigen expression and *in vivo *immune response for recombinant *Salmonella *-HA strains.** (**A**) Expression analysis of the HAOP antigen in the C-HAOP strain using SDS-PAGE with Commassie Blue staining and Western blot. SL7207 was used as negative control. (**B**) Western blot analysis of HAOP expression in the O-HAOP strain, using the HO strain as the negative control. (**C**) Results from ELISA experiments showing HA-specific IgG response raised by the O-HAOP strain using the HO strain as a negative control. 10 mice were used for each group. 1 × 10^11^ bacteria were used to prime each mouse for 3 days, and two boosts were given on day 21 and 35; all by oral gavage. Blood sera from infected mice were collected on days 7, 28 and 42 for ELISA analysis. * underneath Day 42 indicates that the mean ELISA titer raised by the O-HAOP strain was significantly higher than that of the -HAOP strain (*P* < 0.05). (**D**) Results from HIA using blood sera taken from mice infected with O-HAOP strain on day 42. Blood sera from non-infected mice, and mice infected with the HO strain (taken on day 42) were also used a controls. * underneath O-HAOP indicates the mean HIA titer raised by O-HAOP strain was significantly different from those of HO and No-infection groups (*P* < 0.05). (**E**) T cell anti-HA IFN-γ responses provoked by the O-HAOP and C-HAOP strains, using the HO strain as a negative control. Asterisks indicate that the average ELISpot ratios provoked by O-HAOP were significantly different to those provoked by HO or C-HAOP (*P* < 0.05). In (**C**), (**D**) and (**E**), each ▴ represents one mouse. Red lines indicate the average titers in (**C**) and (**D**), and the average ELISpot ratios in (**E**).

For cytoplasmic expression, HAOP was subcloned into the *p*BSK-derived plasmid (*p*HP), placing it under the control of the prosseA promoter (plasmid *p*HAOP). For outer membrane expression, HAOP was subcloned into the *p*HO plasmid to generate *p*O-HAOP. These two constructs were separately transformed into SL7207, to create a cytoplasmic HA expression strain of *Salmonella* (C-HAOP) as well as an outer-membrane HA expression strain of *Salmonella* (O-HAOP). The expression of HAOP in these two recombinant strains was then confirmed by SDS-PAGE and Western blot analysis. As may be seen in Figure 5A, HAOP was highly expressed within the C-HAOP strain, being present almost exclusively in the pellet fraction after cell lysis. This indicates that it may be predominantly localized within insoluble inclusion bodies in the cell cytoplasm. For the strain O-HAOP, Western blot analysis of cell-membrane extracts demonstrated that the expressed HAOP was associated with the outer membrane (Figure 5B).

### *In vivo* immune response to recombinant *Salmonella*-HA strains

#### Humoral immune response

##### Hemagglutination Inhibition Assays (HIA)

HIA tests were performed using blood sera from mice infected with O-HAOP strain on the 42^nd^ day (i.e. after 3 identical immunizations: one prime and two boosts; same schedule as above). Blood sera from non-infected mice, as well as those analogously immunized with the HO (EGFP) strain were used as negative controls. Results (Figure 5D) indicated that sera taken from 50.0% O-HAOP-infected mice (5 out of 10 mice) contained neutralization antibodies with titers ranging from 1:16 to 1:128 (average titer of 1:30). The HIA titers within the O-HAOP-infected mice were significantly different to those obtained for the non-infected group (p < 0.05).

##### Interferon-gamma (IFN-γ) responses to recombinant *Salmonella*-HA strains

As performed for the *Salmonella*-EGFP strains, T cell anti-HA IFN-γ responses raised against the two recombinant *Salmonella*-HA strains (C-HAOP and O-HAOP) were evaluated using ELISpot assays. Non-infected mice as well as HO-infected mice were used as negative controls.

Analogous to before, the anti-HA IFN-γ responses were evaluated by determining the ratio of ELISpot numbers counted for the C-HAOP, O-HAOP or HO infected mice, divided by the number counted for the non-infected control set (Figure 5E). It was found that the C-HAOP strain could not induce an anti-HA IFN-γ response, with an average ratio of 0.91 obtained, *versus* 0.69 determined for the HO-infected group. However, the O-HAOP strain elicited a better anti-HA IFN-γ response comparing to the HO and C-HAOP groups (p < 0.05), yielding an average ratio of 2.56.

## Discussion

### B cell responses and soluble antigen expression levels

The use of recombinant *Salmonella* strains as live attenuated vaccines has been attracting increasing interest over recent years, due to the low cost and relative ease by which they may be produced on a large scale. [23-25]. Although numerous *Salmonella* vaccine systems have been developed, relatively little work has been carried out to systematically investigate which approaches or construction methods reproducibly produce recombinant strains with the highest immunogenicity levels. Here, we used EGFP as a model antigen to study the relationship between immunogenicity and the use of different antigen expression systems in *Salmonella*. Six recombinant strains, including three cytoplasmic expression strains (HP, LP and CP); two outer-membrane expression strains (HO, CO) and one eukaryotic expression plasmid (strain E), were constructed. Several different pairs of EGFP-expression systems were combined, to produce five additional ‘double-recombinant’ strains. The antigen expression levels; *in vitro* and *in vivo* growth and infection abilities; and the immunogenic properties of these single and double-recombinant strains in mice; were systematically investigated, in order to evaluate their respective advantages, and to identify deleterious or synergistic effects.

Within the set of three single-recombinant strains designed for cytoplasmic expression of antigen protein, the high copy plasmid *p*HP produced the highest amounts of EGFP within the HP cells *in vitro* (Figure 2B); resulting in relatively strong B cell responses (anti-EGFP IgG response rate, Figure 4A), even though the plasmid itself did not exhibit good stability *in vitro and invivo* (Table 4 and Figure 3). Although the EGFP expression levels in the LP strain (containing the low-copy *p*LP plasmid) were lower than those in the HP strain, LP elicited a better B cell response, which is probably due to high EGFP expression and improved plasmid stability in *vitro and in vivo*. The CP strain, which contains a (single-copy) chromosome-based expression cassette, produced lower levels of EGFP expression than the HP and LP strains, and induced a weaker B cell response rate than these two strains. Out of the two single-recombinant strains designed for outer membrane expression, the HO strain, which harbors the high copy number *p*HO plasmid, expressed higher levels of EGFP than the chromosome-based CO strain *in vitro*, and also elicited better B cell response rate. The EGFP expression levels in the HO strain were lower than those in HP or LP, the high copy plasmid *p*HO showed poor *in vivo* stability (as did *p*HP), and it also induced a weaker B cell response than the LP strain. The B cell response rate against the E strain, which harbors a eukaryotic expression plasmid, were weaker than those raised by the HP, LP and HO strains; most likely because the EGFP cannot be expressed until *p*EGFP has entered a host cell. In addition, the eukaryotic plasmid expression levels may not be as high as those generated by *Salmonella* strains containing high or low copy prokaryotic plasmids. Taken together, these findings suggest the strength of the humoral immunity responses in mice infected with recombinant *Salmonella* vaccines were positively correlated to antigen expression levels and plasmid stability. Therefore, maintaining high and stable antigen expression levels in recombinant *Salmonella* strains is of key importance for eliciting a strong B cell response. Furthermore, our results indicate that strains containing low copy prokaryotic plasmids are capable of generating highest B cell responses with relative high antigen expression level and improved stability over high-copy plasmids.

During previous efforts to develop live recombinant *Salmonella* oral vaccines, the expression of heterologous antigens within *Salmonella* cells was achieved mainly through the use of multi-copy prokaryotic plasmids which contain the antigen of interest under the control of a prokaryotic promoter. Plasmids with various copy numbers have been used [26,27]. In several cases, such as the construction of live attenuated *Salmonella* oral vaccines against *Streptococcus pneumonia*, the H5N1 virus or hepatitis B virus; the antigens of interest expressed from high copy prokaryotic plasmids were able to induce strong immune responses, especially humoral responses [7-9]. However, many of these vaccine strains had either negligible or fairly low immunogenicity, due to the instability of the high copy plasmid, or toxic effects induced by the unregulated high-level expression of these heterologous antigens [28-30]. Plasmids may be stably maintained in *Salmonella* cells during *in vitro* growth via the use of the appropriate antibiotic. However, the loss of high copy plasmids may easily occur once *Salmonella* infects and grows inside the host, due to the absence of antibiotic selection pressure; thereby resulting in reduced antigen expression levels and decreased immunogenicity *in vivo*. Also, the unregulated high level expression of antigen may create a significant metabolic burden, or even toxicity for the *Salmonella* cell, thus creating a selective pressure for high copy plasmid segregation during cell division [27]. Here, the LP strain, which contained a low copy plasmid, exhibited relatively high EGFP expression levels with improved *in vitro* and *in vivo* stability, compared with strains containing high copy plasmids. This strain induced the highest levels of B cell immunogenicity (Table 4 and Figures 2B, 3 and 4). This result is similar to those reported in previous studies, which have found that lowering the plasmid copy number results in enhanced plasmid stability and a reduction in the expression levels of heterologous antigens to non-toxic levels [31-34].

Problems associated with plasmid instability and toxicity due to high-level antigen expression may be circumvented through the use of a chromosome-based expression strategy. In this and other studies, homologous recombination has been used to insert a heterologous antigen expression cassette into the *Salmonella* chromosome, resulting in the creation of recombinant strains with good *in vitro* and *in vivo* stabilities (Table 4 and Figure 3) [35,36]. However, as the heterologous gene is present as a single copy per bacterium, the expression levels may be too low to effectively induce a B cell response. In this study, the poor B cell responses against the chromosome-based expression strains CP and CO were probably due to the low levels of EGFP expression (Figures 2B and 4A). Similar findings have been previously reported. For example, during efforts to develop a live *Salmonella* oral vaccine against fragment C of the tetanus toxin (TetC), the recombination of TetC into the *aro*C gene of *S.* typhimurium created a strain with good stability; however low TetC expression levels resulted in poor immunogenicity. One possible way to increase expression levels would be to insert multiple copies of the heterologous antigen genes into chromosome.

### T cell responses, eukaryotic expression and colonization with good stability

*Salmonella* has been used as a delivery vector for eukaryotic plasmids for more than ten years [37-40]. In these DNA vaccines, the plasmid encoding the target antigen gene is placed under the control of a eukaryotic promoter. Theoretically, *Salmonella* delivered-mucosal DNA vaccines can invade the Peyer’s patches via M cells in the intestinal mucosa, which contain many antigen-presenting cells (APCs); such as macrophages and dendritic cells [41]. Once phagocytized by the APCs, the recombinant *Salmonella* cells would be lysed within the phagosomes, releasing the eukaryotic plasmids into the APCs. The APCs would then express the plasmid-encoded antigen and present them to CD4^+^ or CD8^+^ T cells in the Peyer’s patches or mesenteric lymph nodes via MHC I and MHC II molecules. In addition, the APCs may directly secrete the recombinant antigens from the cells, so that B cells can recognize them via B cell receptors (BCRs). This process induces further mucosal, humoral or cellular immune responses against the heterologous antigen [38,42-45]. Many studies have found that *Salmonella* delivered DNA vaccines were particularly good at generating T cell responses against foreign antigens. In this project, we constructed a *Salmonella*-DNA vaccine strain (strain E) which contains a high copy eukaryotic plasmid (*p*EGFP). Using Western blotting, we confirmed that *p*EGFP plasmid delivered by the E strain could be used to successfully express EGFP in mice intestinal mucosal sites on day 1 after inoculation (Figure 1E). Furthermore, we found that this *Salmonella*-eukaryotic plasmid-based system could induce better T cell responses than the *Salmonella*-high-copy-prokaryotic plasmid-based systems (Figure 4C); However, its antibody responses were quite low (Figure 4A). Analogous findings have previously been reported. In one comparative study, it was shown that an oral *Salmonella* DNA vaccine strain encoding β-galactosidase induced better T helper cell and CTL responses than oral immunization with a *Salmonella* strain containing a prokaryotic plasmid-encoded β-galactosidase gene [38]. Similarly, oral immunization with the surface protein from the hepatitis B virus delivered by a *Salmonella* DNA vaccine elicited good T cell responses, but its B cell responses were quite low [46]. Based on our and others’ comparative analyses, we consider it likely that the endogenously produced antigen delivered by the *Salmonella*-DNA vaccine may generate immune responses mainly through the MHC I pathway, thus resulting in higher T cell responses but lower B cell responses.

ELISpot results suggested that a chromosome-based expression system (CP strain) could also generate better T cell responses than strains containing high copy prokaryotic plasmids. Furthermore, results from the *in vivo* survival, infection ability and construct stability assays (Figure 3), indicated that the CP strain had better survival abilities and construct stability than strains containing high copy plasmids. These findings indicated that colonization with recombinant vaccine strains with good construct stability is also an important factor for eliciting strong T cell responses. This is most likely due to the following reasons: T cell responses against *Salmonella* infection can be effectively induced only after the recombinant cells have invaded and proliferated within the APCs [47]. However, after oral administration, the bacterial cells would be in a low-nutrient environment that lacked antibiotic selective pressure. They would also be subjected to acidic conditions within the GI tract (even if this was partially negated by sodium bicarbonate lavage) as well as attack by the host immune defenses. Consequently, non-integrating (episomal) plasmids would be lost fairly rapidly as observed in survival, infection ability and construct stability analyses (Figure 3), thereby greatly reducing the abilities of the bacterium to synthesize antigen protein *in vivo*. Chromosome-based strains such as CP avoid this problem, albeit at the price of reduced antigen expression levels. Even though the chromosome-based CO strain showed *in vivo* stabilities equivalent to the CP strain, its T cell responses were lower. It is possible that the membrane-expressed EGFP significantly slowed the growth rate of the CO strain (Table 2), resulting in poorer *in vivo* survival and proliferation abilities (colonization ability), compared to the CP strain (Figure 3A).

It is notable that for the various single-recombinant strains, the general trend observed for the T cell responses ran contrary to the trend observed for the humoral immunity responses (described above). In this regard, the properties of the LP strain are worth commenting on. Even though its induced T cell responses were not as high as those elicited by the E and CP strains, it could simultaneously induce both B cell and T cell responses. It also exhibited better *in vivo* stability than the strains containing high copy plasmids (Figure 3). Consequently, if the aim was to develop a vaccine strain capable of inducing both B cell and T cell responses, then the use of a low copy cytoplasmic expression system may be desirable.

### Immunogenicity of strains encoding two EGFP expression cassettes

Since the strains containing the high or low copy prokaryotic plasmids (HP, LP, HO) could induce better B cell responses, while strains containing the eukaryotic expression plasmid (E) or the chromosome-based expression cassette (CP) elicited higher anti-EGFP IFN-γ responses, we hypothesized that a combination of two individual expression strategies may result in both stronger B and T cell responses. Consequently, we created five double-recombinant strains (CP + HP, CP + LP, CP + HO, CP + E, E + LP). We used CP and E as the starting strains for the construction of these five dual-expression systems, using compatible plasmid replication origins and orthogonal antibiotic resistance genes. The CP + HP, CP + LP, CP + HO and E + LP strains were designed to elicit both stronger B cell and T cell responses. The CP + E strain was constructed to investigate whether it could generate a T cell response that was higher than those of the two individual strains. Contrary to our initial expectations, both the humoral and T cell immune responses against these five double-recombinant strains were not markedly improved, some of them were obviously worse than their individual parent strains (Figure 4). There are several possible reasons for this lack of synergy: first, the combination increased the frequency of plasmid loss. According to our *in vitro* plasmid stability studies (Table 5), we found that the detection rates for *p*HP in the CP + HP strain, *p*HO in the CP + HO strain and *p*EGFP in the E + LP strain were 0.8%, 60.0% and 50.0%, respectively. This suggested that the high copy plasmids were more easily displaced in the double-recombinant strains, resulting in a decrease in EGFP expression levels (Figure 2B) and B cell responses (Figure 4A and B). Second, antibiotic selection pressure was increased. To maintain the plasmids, we used relatively high concentrations of two antibiotics during the subculturing procedures. However, the high-copy plasmids, containing *p*UC replication origins, were still frequently lost from the cells. Upon losing the plasmid, the relevant antibiotic will become toxic to the cell, resulting in significantly decreased growth rates for the five double-recombinant strains (Tables 2 and 3). It would also cause an increase in ‘stress’ for the cell, resulting in defects in replication and other growth-related effects; which were manifested in the frequent formation of filamentous cells (Figure 2A) [48-50]. Furthermore, even though the high-copy plasmids were not lost from some of the recombinant *Salmonella* strains *in vitro*; as was noted for the HO, E and CP + E strains (Tables 4 and 5), cell elongation was still observed (Figure 2A). This may be due to the ‘metabolic burden’ [51-53] caused by the active maintenance of multiple high copy plasmids within the cell, leading to decreases in its overall immunogenic properties.

### Expression strategies for insoluble antigens in *Salmonella* oral vaccine strains

As the expression abilities of *Salmonella* are relatively similar to those of *Escherichia coli*, this prokaryotic system may not be suitable for the expression of protein antigens from viruses or higher eukaryotes [38]. Consequently, when constructing a vaccine strain against a virus such as avian influenza H5N1, several factors must be taken into consideration. The first is codon bias; however this may be easily resolved by gene synthesis, using the codons preferred by *Salmonella*. The second problem is the inability to express soluble antigen within the cytoplasm, or to target it to the cell surface in a correctly folded form. Many antigens from eukaryotic viruses, such as the HA protein from H5N1, cannot be readily expressed in a soluble form within the cytoplasm of *Salmonella*[8]. This type of situation can dramatically decrease the B cell and T cell immune response within the infected host. According to our *in vivo* immune response assays, no detectable B cell or T cell responses could be detected when mice were immunized with the C-HAOP strain, which was designed to cytoplasmically express the HA antigen.

It has previously been reported that expressing the antigen protein on the outer membrane of the engineered bacterial cells may increase the immune response [54], since this may increase the likelihood of recognition by the host immune system. To investigate this issue, we targeted the EGFP protein for outer membrane expression using the lpp-ompA outer membrane expression system. Analogous outer membrane expression cassettes were located on a high-copy plasmid (HO strain) and on the chromosome (CO strain). The B cell responses (Figure 4A) against these two strains were weaker than those obtained for the LP strain; and the T cell responses (Figure 4C) against the HO strain were lower than those obtained for the E and CP strains. This may be because the total amounts of EGFP synthesized and exported to the outer membrane were too low to induce an effective B cell response (Figure 2B), or because the *in vivo* stabilities of the high copy plasmids were not sufficient to induce an effective T cell response (Figure 3). However, this strategy proved somewhat more successful for the HA antigen, which appears to be largely present in insoluble inclusion bodies in the cytoplasm of *Salmonella*. We found that the use of the lpp-ompA system, which is present in the O-HAOP strain, resulted in the successful export of HA antigen to the outer membrane fraction (Figure 5B). Furthermore, results from ELISA, HIA and ELISpot assays indicated this strain of *Salmonella* was capable of inducing better HA-specific B cell and T cell responses than the corresponding C-HAOP strain, which expresses the HA antigen in the cytoplasm (Figure 5C, D and E). It is possible that the use of the Lpp-OmpA system either helps the HA protein fold into a soluble conformation, or results in the export of increased amounts of HA protein, causing notable enhancements in immunogenicity.

## Conclusions

In this study, we used EGFP and HAOP as model antigens to systematically compare the immunogenic potency of various recombinant *Salmonella* strains as live, oral antigen-delivery vectors. Our results indicate that if the antigen (such as EGFP) is soluble and easily expressed in *Salmonella*, a low-copy plasmid-based strategy should be employed, as it can provoke better B cell responses and can also induce T cell responses. If a T cell response is preferred, a eukaryotic plasmid, or chromosome-based expression strategy may achieve better results. For heterologous antigens that are likely to be expressed in an insoluble form within *Salmonella* (such as HA), an outer membrane-targeting approach is recommended. In addition, we found that the combination of two expression strategies did not enhance the immune response, and hence we caution against the use of such an approach.

## Methods

### Ethics statement

All animal work was conducted according to relevant national and international guidelines (Animal Research: Reporting *In Vivo* Experiments guidelines). 6-8-week-old female BALB/c mice purchased from the Laboratory Animal Unit of the University of Hong Kong were used for this study. This study was approved by the Committee on the Use of Live Animals in Teaching and Research (CULATR) of The University of Hong Kong, with the CULATR Number 2051–09.

### Bacterial strains, media, chemicals, enzymes and plasmids

*Escherichia coli* strain DH10B (Invitrogen) was used for all plasmid construction experiments. Attenuated *Salmonella enterica serovar* Typhimurium *aroA*^-^ strain SL7207 was kindly provided by Dr. B.A.D. Stocker [55]. All bacteria were cultured in Luria-Bertani (LB) broth at 37°C. Minimal medium for target protein induction was based on the N-salts recipe [56]. Antibiotics (Sigma) for clone selection were used at the following final concentrations: ampicillin (Amp, 200 μg/ml), kanamycin (Km, 50 μg/ml) and chloramphenicol (Cm, 25 μg/ml). Restriction endonucleases and ligase enzymes were purchased from New England BioLabs (NEB). *Taq* polymerase (*Pyrobest* and *Ex-taq*) and the pMD18-T vector were from Takara. Plasmid *p*Sim6 was a gift from Dr. D.L. Court. Plasmid *p*Bluescript II SK (*p*BSK) was bought from Stratagene. Other plasmids were from lab stocks.

### Construction of recombinant *Salmonella* strains expressing model antigens

#### Construction of ‘single-recombinant’ *Salmonella*-EGFP strains

The compositions of six ‘single-recombinant’ *Salmonella*-EGFP strains (i.e. strains containing a single episomal or chromosomal copy of the EGFP gene) are listed in Table 1. The primers used to construct the six ‘single-recombinant’ *Salmonella-*EGFP strains are listed in Table 6. The *prosseA* promoter was PCR amplified from pathogenicity island 2 (SPI2) of SL7207 using the NotI-prosseA-F and HindIII-prosseA-R primers; the EGFP gene was PCR amplified from *p*loxp-cm-loxp with the HindIII-EGFP-F and XhoI-Ter-EGFP-R primers; then these *prosseA* and EGFP fragments were co-ligated into vector *p*BSK via NotI and XhoI to create *p*HP.

**Table 6:** **Primers used in this study**

**Primer Name**	**Primer Sequence (5’ to3’)**
NotI-prosseA-F	ATTTGCGGCCGCAGAAGAGAACAACGGCAAGTTAC
HindIII-prosseA-R	CCCAAGCTTACGATAGATAATTAACGTGC
XhoI-floxed-F	CCGCTCGAGCCGATCATATTCAATAACCCT
XhoI-floxed-R	CCGCTCGAGGACTAGTGAACCTCTTCGAGGG
HindIII-EGFP-F	CCCAAGCTTAAGAAGGAGATATACATATGGTGAGCAAGGGCGAGGAGC
XhoI-Ter-EGFP-R	CCGCTCGAGCGGCCGCAAAAAACCCCTCAAGACCCGTTTAGAGGCCCCAAGGGGTTATGCTAGTTACTTGTACAGCTCGTCCATGCC
*p*15A-F	TATCACTTATTCAGGCGTAGCACC
*p*15A-R	ATCGTATGGGGCTGACTTCA
ompA-F	GGGAATTCCATATGAAAGCTACTAAACTGGTACTGGGCGCGGTAAACCCGTATGTTGGCTTTGAAATGGG
ompA-R	CCGCTCGAGTTATGCGGCCGCGTTGTCCGGACGAGTGCCGATGGTGT
oEGFP-F	ATTTGCGGCCGCAGTGAGCAAGGGCGAGGAGC
oEGFP-R	CCGCTCGAGTTACTTGTACAGCTCGTCCATGCC
htrA-F	CGCGTTATAAAATGAATCTGACGTACACAGCAATTTTGCGTTACCTGTTAATCGAGATTGAAACACAGGGTTTTCCCAGTCACGACGTT
htrA-R	AGTTGTGGGGAGTTTCACAGAAAAGTGTTGCCCCCTTCCGTGGTGGAAGGGGGACAAAGGTGATTACTGGAGCGGATAACAATTTCACACAGG

The *p*15A origin was amplified from plasmid *p*ACYC177 using primers *p*15A-F and *p*15A-R, and an *EGFP* expression cassette was amplified from *p*HP using primers NotI-prosseA-F and XhoI-Ter-EGFP-R. Both fragments were directly ligated together to creat *p*LP.

The lpp*-*OmpA outer membrane expression cassette was amplified from SL7207 using primers ompA-F and ompA-R, and was ligated into the *p*MD18-T vector to create plasmid *p*OmpA. *p*HO was constructed by ligating an EGFP cassette (amplified from *p*loxp-cm-loxp using primers oEGFP-F and oEGFP-R) downstream of lpp-OmpA in *p*OmpA via NotI and XhoI. Strains HP, LP, HO and E were generated by transforming plasmids *p*HP, *p*LP, *p*HO and *p*EGFP, respectively into SL7207 by electroporation.

A loxp-Cm-loxp cassette was PCR amplified from plasmid *p*loxp-Cm-loxp using primers XhoI-floxed-F and XhoI-floxed-R [57], digested with XhoI, then ligated into the XhoI site of plasmid pHP to create plasmid *p*CP-cm. *p*CO-cm was generated by replacing the EGFP gene in *p*HP with *NdeIlpp-OmpA-EGFPXhoI* and *XhoI-loxp-Cm-loxp-XhoI* fragments. The linear dsDNA fragments used to create the CP and CO chromosome-based expression strains were respectively amplified from *p*CP-cm and *p*CO-cm by PCR using a same pair of primers (htrA-F and htrA-R). Red recombineering was used to construct the CP and CO strains, via transient expression of λ-Red genes from *p*Sim6 in SL7207 according to the method of Yu *et al.*[22,58]. Transformants were selected by plating onto LB agar plates containing the required antibiotics, incubating at 37°C overnight.

#### Construction of the ‘double-recombinant’ *Salmonella*-EGFP strains

The compositions of the five ‘double-recombinant’ *Salmonella*-EGFP strains (i.e. strains containing two individual copies of the EGFP gene) are described in Table 1. Strains CP + HP, CP + LP, CP + HO and CP + E were generated by respectively electro-transforming plasmids *p*HP, *p*LP, *p*HO or *p*EGFP into the CP strain. The LP + E strain was generated by electro-transforming *p*EGFP into the LP strain. Successful transformants were selected on LB agar plates containing the appropriate antibiotics, incubating at 37°C overnight.

#### Construction of two recombinant *Salmonella*-HA strains

The sequence of the HA epitope (residues 91–261) from the H5N1 virus (H5N1/A/Vietnam/1194/2004) was codon-optimized for translation in *Salmonella* based on sequence data from the NCBI-GenBank, and synthesized by GenScript (Piscataway, USA). This synthetic codon-optimized HA epitope gene (denoted HAOP) was cloned between the NotI and XhoI sites of *p*HP (thereby replacing the EGFP gene) to create *p*HAOP. *p*O-HAOP was analogously generated by replacing the *EGFP* gene of *p*HO with a NotI/XhoI-digested HAOP fragment. Plasmids *p*HAOP and *p*O-HAOP were respectively electro-transformed into SL7207 to generate a cytoplasmic-expression HAOP *Salmonella* strain (denoted C-HAOP) and a membrane-expression HAOP *Salmonella* strain (denoted O-HAOP).

#### Culture of recombinant *Salmonella* strains

In order to minimize plasmid loss, the recombinant *Salmonella* strains were freshly streaked from −80°C stocks onto LB agar plates containing the required antibiotics (Table 1) and incubated at 37°C overnight. Single colonies were inoculated into 30 ml liquid LB medium with antibiotics and cultured at 37°C with shaking for 10 hours. The 10-hour bacterial cultures were expanded (1:10) into fresh LB medium containing antibiotics, before being further incubated at 37°C until OD_600_ = 2. With the exception of the CP and CO strains, cells were then collected by centrifugation (4,000 g, 4°C); washed once with PBS; resuspended in PBS equal to the volume of the original culture; then immediately used for immunization or expression analysis. Further induction was required for the CP and CO strains, using equal volumes of N-salts medium. Prior to induction, freshly-cultured CP or CO strains were collected by centrifugation (4,000 g, 4°C), then washed twice with N-salts medium in equal volume of bacteria cultures. Cells were then resuspended in N-salts medium containing the appropriate antibiotics (equal to the original volume of the bacterial culture), and incubated at 37°C for 24 hours to induce protein expression. After 24 hours, cells were collected for expression analysis or immunization as above.

### Expression analysis of recombinant *Salmonella* strains

#### Fluorescence microscopy

Fluorescence microscopy (Olympus BX51 microscope) was used to analyze EGFP expression within the eleven recombinant *Salmonella* strains. Freshly-cultured bacterial cells (10 μl) were immobilized on 1% agarose pads on glass slides, then visualized using a 60x oil-immersion objective lens. Both bright-field and fluorescent field (ex. 488 nm, em. 518 nm) images were captured (Spot RT3 Digital Camera, Spot Advanced Software Version 4.6). Freshly cultured SL7207 cells were used as a negative control.

#### Fluorescence intensity assays

A fluorescence micro-plate reader (Varioskan Flash, Thermo Fisher Scientific) was used to quantify EGFP expression levels within freshly-cultured recombinant *Salmonella* strains. 1 × 10^8^ cells were resuspended in 100 μl PBS in 96-well plates (IWAKI, 96 well microplate with flat bottom), and EGFP fluorescence intensity was immediately recorded at room temperature (ex. 518 nm, em. 518 nm; time resolution: 500 ms). Measurements were performed in triplicate, and SL7207 was used as a negative control.

#### SDS-PAGE and Western blot analysis

Sodium dodecyl sulfate-polyacrylamide gel electrophoresis (SDS-PAGE), followed by Western blot analysis was performed to detect EGFP expression in the CO strain. The *in vitro* and *in vivo* EGFP expression levels in E, HAOP, C-HAOP and O-HAOP strains; were determined using slightly-modified protocols for each strain.

*CO strain*: cells from 4 ml of induced bacterial culture were collected and resuspended in 100 μl PBS + 50 μl of loading buffer (60 mM Tris–HCl, pH 6.8, 25% glycerol, 2%SDS, 14.4 mM β-mercaptoethanol, 0.1% bromophenol blue) and boiled (10 minutes) to prepare lysates for analysis. GFP rabbit polyclonal IgG (Santa Cruz Biotechnology) and goat anti-rabbit antibody conjugated with horseradish peroxidase (Invitrogen), were used as the as primary and secondary antibodies, respectively. SL7207 cells were also treated as above as the negative control.

*E strain*: *in vitro* and *in vivo* EGFP expression was quantified by Western blot. The EGFP expression in the eukaryotic cells was first detected *in vitro* by infecting the Caco-2 human colon carcinoma cell line (American Type Culture Collection) with the E strain. The Caco-2 human colon carcinoma cell line was cultured using Dulbecco’s modified eagle’s medium (DMEM) (Invitrogen) with 20% fetal bovine serum (FBS) (Invitrogen) and 1% penicillin/streptomycin (P/S) (Invitrogen) in 24-well plate (TPP, USA) with 5 × 10^5^ cells per well at 37°C in 5% CO2. After cell confluence reached 90%, the medium was removed and the cells were washed with PBS twice. Then PBS-washed 1 × 10^7^ CFU bacterial cells of E strain in DMEM-20% FBS in the absence of antibiotics were used to incubate with the Caco-2 cells. After 2.5 hours incubation, the non-adherent bacteria were removed by washing with PBS three times. DMEM with 20% FBS was added to maintain the cells at 37°C in 5% CO_2_. Cell samples were collected 12, 20 and 30 hours after infection, respectively. Caco-2 cell protein was extracted using RIPA buffer (50 mM Tris–HCl pH 7.4, 150 mM NaCl, 1 mM EDTA, 0.25% Deoxycholate, 1% NP40) plus protease inhibitor cocktail tablets (Roche). Briefly, cells were harvested and washed with cold PBS twice (2500 x g, 5 min), then RIPA buffer containing protease inhibitor was added to mix the cell pellet completely, and this was incubated at 4°C for 15 minutes. The RIPA/cell mixture was centrifuged (13000 x g, 1 min) and the supernatant was collected for Western blot assay. The protein quantities were measured using the BCA protein assay kit (Thermo Fisher Scientific Inc). Equal amounts of protein from the samples were loaded to detect EGFP expression by Western blot using a GFP monoclonal antibody (Santa Cruz Biotechnology) and a goat anti-mouse secondary antibody conjugated with horseradish peroxidase (DAKO). GAPDH detection was also performed using an HRP conjugated antibody (Abnova). The SL7207 strain was also treated as above as the negative control.

EGFP expression delivered by the E strain was also investigated in the mouse intestine. 1 × 10^11^ of bacterial cells of the E strain or the SL7207 strain re-suspended in 200 μl PBS, were orally administrated to 6-8-week-old female BALB/c mice after neutralizing the gastric acid (gavage with 100 μl of 3% sodium bicarbonate), respectively. Intestinal mucosa samples from two groups of mice were collected using heated glass slides on day 1, 3 and 7 after inoculation. Total proteins of intestinal mucosa were extracted using RIPA buffer plus protease inhibitor as mentioned before. Equal amounts of protein samples quantified by BCA protein assay were used to perform Western blot, to detect EGFP expression using the same protocol mentioned above. GAPDH was also detected as the internal control.

*C-HAOP strain*: total cell lysate (from 1 × 10^9^ bacterial cells) was prepared as described above. For the cell-fraction lysates, 1 × 10^9^ cells were resuspended in 100 μl PBS containing protease inhibitors (Roche, Complete EDTA-Free), then sonicated (Sonics, model VCX750; with ice cooling). After centrifugation (12,000 x g; 1 min; 4°C), the supernatant (ca. 90 μl) was decanted and 50 μl of (x3) loading buffer added. The pellet was resuspended in 100 μl of (x1) loading buffer. Samples were similarly boiled (10 mins), prior to Western blot analysis using rabbit polyclonal influenza A virus hemagglutinin antibody (ab36565, Abcam) and goat anti-rabbit antibody conjugated with horseradish peroxidase (Invitrogen) as primary and secondary antibodies, respectively. SL7207 cells were treated as above as negative control.

*O-HAOP strain*: 40 ml of freshly-cultured bacterial cells were harvested by centrifugation (3,500 x g, 15 mins, 4°C), resuspended in 10 ml PBS containing protease inhibitors, then sonicated 4°C). The cell debris was removed by centrifugation (3,500 x g, 10 mins, 4°C), then the supernatant was ultra-centrifuged (100,000 x g, 60 mins, 4°C) to collect the total membrane fraction (as the pellet). A portion of the membrane fraction was resuspended in PBS containing 0.01 mM MgCl2 and 2% Triton X100, and then centrifuged (100,000 x g, 60 mins, 4°C). The inner membrane fraction was collected as the supernatant; whilst the pellet, which contains the outer membrane fraction, was resuspended in 1 ml PBS. The protein concentration of each fraction was measured using the bicinchoninic acid (BCA) protein assay (Thermo Fisher). Each fraction was boiled with loading buffer (as above) and analyzed by Western blotting using rabbit polyclonal influenza A virus hemagglutinin antibody and HRP-conjugated goat anti-rabbit antibody as primary and secondary antibodies, respectively. The membrane fraction from the HO strain was used as a negative control.

#### Morphological observation of recombinant *Salmonella*-EGFP strains

Microscopic images of SL7207 and the various recombinant *Salmonella*-EGFP strains were captured using light microscopy (Olympus BX51 microscope). Twenty cells from each recombinant strain, as well as the SL7207 control were randomly selected from the microscopic images for cell length analysis using the Image J software program [58]. The mean cell length for each strain was reported ± standard deviation and then compared to the SL7207 control.

#### *In vitro* growth of recombinant *Salmonella*-EGFP strains and stability of EGFP expression constructs under antibiotic pressure

To assess the growth of the six single-recombinant and five double-recombinant *Salmonella*-EGFP strains *in vitro*, the doubling times of these eleven recombinant strains were measured in liquid LB broth with antibiotics. Apart from using SL7207 as a general negative control, the empty vectors: *p*BSK, *p*ACYC177, *p*OmpA, *p*VAX-1 were electro-transformed into SL7207 to create strains: BSK, ACYC177, OmpA and VAX-1 as the negative controls of HP, LP, HO, E strains, respectively. The VAX-1 + ACYC177 strain was generated by co-electrotransforming *p*ACYC177 and *p*VAX-1 into SL7207, as a negative control for the E + LP strain. 10-hour bacterial cultures from each strain obtained using the protocol described in Part 3 were diluted 1:100 into fresh LB medium containing antibiotics. The diluted cultures were then incubated at 37°C with shaking. Bacterial growth was measured spectrophotometrically using OD_600_, at intervals of 30 mins. Four replicates were performed for each strain, and the mean is reported ± standard deviation.

The stability of the six single-recombinant and five double-recombinant *Salmonella*-EGFP strains under antibiotic selective pressure, prior to oral administration were measured via colony forming unit (CFU) assay. 1 × 10^9^ cells from each strain were collected and resuspended in 1 ml fresh LB. Six 1:10 serial dilutions were performed, and 10 μl of each dilution was spread on LB agar plates, or LB agar plates containing the appropriate antibiotics as listed in Table 1. Colonies were counted after incubation overnight at 37°C. Three replicates were performed for each dilution, and the mean is reported ± standard deviation.

#### *In vivo* survival and infection ability of six single-recombinant *Salmonella* strains and stability of EGFP expression constructs

In order to determine the survival and infection abilities of the six single-recombinant *Salmonella* EGFP strains, and the *in vivo* stabilities of the six EGFP expression constructs, 1 × 10^11^ of freshly cultured bacterial cells from each of the six strains resuspended in 200 μl PBS, were separately orally administrated to 6-8-week-old female BALB/c mice after neutralizing the gastric acid. 6 mice were grouped for each of the six strains, and one group of mice immunized with SL7207 was kept identically as the negative control. Fecal, spleen and mesenteric lymph nodes samples were collected from each group on days 1, 3 and 7 after administration for CFU assays, respectively. All the samples were homogenized and re-suspended in 9 × LB broth according to sample weights. Then seven 1:10 serial dilutions were carried out in a final volume 100 μl. 10 μl of each dilution was spread on LB agar plates, or LB agar plates containing the appropriate antibiotics as listed in Table 1. Enumeration was performed after incubation overnight at 37°C. Three replicates were performed for each dilution, and the mean is reported ± standard deviation.

### *In vivo* immune response assays

#### Animal immunization

6-8-week-old female BALB/c mice purchased from the Laboratory Animal Unit of the University of Hong Kong (CULATR 2051–09) were used for all *in vivo* immune response tests. Identical batches of 1 × 10^11^ of PBS-washed, freshly-cultured bacterial cells resuspended in 200 μl PBS were used for each immunization procedure (prime and boost) for each strain. Immediately prior to oral administration, each mouse was orally gavaged with 100 μl of 3% sodium bicarbonate to neutralize gastric acid. Cells were administered by oral gavage; giving two boosts on day 21 and day 35 after the initial prime. Groups of 5–10 mice were used for each of the 11 recombinant *Salmonella*-EGFP strains, and one group of non-infected mice were kept identically as the negative control. In analogous parallel experiments, three further groups of 5–10 mice were orally immunized with the C-HAOP, O-HAOP or HO (as control) strains; with an additional group of non-infected mice kept as a negative control.

### Humoral immunity assays

#### ELISA

Enzyme-linked immunosorbent assays (ELISA) were used to detect antigen-specific IgG response using mice serum on day 7, 28 and 42. ELISA plates (Ni-NTA HisSorb^TM^ plate, QIAgen) were pre-coated with 100 ng GFP (Millipore) or H5 antigen (H5N1, A/Vietnam/1203/2004, Immune Technology Corp.) in 100 μl of PBS containing 0.2% BSA, at 4°C overnight. The pre-coating solution was removed, then unreacted sites were blocked with 200 μl of PBS containing 1% BSA, incubating for 2 hours at room temperature. After removal of blocking buffer, serial dilutions of mice serum in 100 μl of PBS containing 0.2% BSA were added, then plates were incubated at room temperature for 2 hours. Unbound serum antibodies were washed-off with PBST (6 washes). Goat anti-mouse IgG antibody conjugated with horseradish peroxidase (Invitrogen, 20,000-fold dilution in 100 μl of PBS containing 0.2% BSA) was added and plates were incubated at room temperature for 1 hour. After 6 washes with PBST, 200 μl of TMB solution (Calbiochem) was added, and plates were incubated at room temperature until the appearance of a blue color. The reaction was stopped by adding 50 μl of 2 M sulphuric acid, then intensities were determined at 450 nm using a microplate reader (Spectra Max 340, GrandTech Scientific).

#### Hemagglutination inhibition assay (HIA)

Hemagglutination inhibition assays (HIA) were performed according to a standard protocol with minor modifications [59]. Briefly, serial two-fold dilutions of O-HAOP vaccinated mice serum (ranging from 1:8 to 1:256, in PBS; final volume of 25 μl;) were added to wells of a microtiter plate, together with 25 μl of H5N1 virus suspension (H5N1/A/Vietnam/1194/2004). Microplates were incubated at room temperature for 30 minutes with gentle shaking. 50 μl of chicken red blood cell suspension was added to each well, and plates were incubated at room temperature with gentle shaking until the chicken red blood cell control (chicken red blood cell in 50 μl PBS) formed a button at the bottom of a well. The HIA titer for each serum sample was defined as the highest dilution of serum that could completely inhibit hemagglutination. Sera from non-infected mice, as well as those inoculated with the HO strain were used as negative controls. Three replicates were performed for each dilution.

#### ELISpot

ELISpot assays were used to detect the antigen-specific mouse IFN-γ response as described in the Mabtech manual of ELISpot kit. The ELISpot plate (Mabtech) was first activated by adding 50 μl of 70% ethanol to each well for 2 minutes, followed by washing 5 times with 200 μl sterile water. Wells were then pre-coated with 100 μl of 15 μg/ml anti-mouse IFN-γ antibody (AN18 Mabtech) diluted in sterile PBS, incubating at 4°C overnight. Excess antibody was then removed by washing 5 times with 200 μl sterile water. Plates were incubated with 200 μl of RPMI 1640 medium containing 10% fetal bovine serum (FBS) for 30 minutes. During incubation, spleens taken from day 42 mice were homogenized and treated with red blood cell lysis buffer (10 mM KHCO_3_, 150 mM NH_4_Cl, 0.1 mM EDTA). The spleen cell homogenates were filtered (40 μm cell strainer, BD Falcon) then centrifuged (1000 x g, 8 minutes, 4°C). Cell pellets were suspended in RPMI 1640 medium containing 10% FBS. 2 × 10^5^ cells in 100 μl RPMI 1640 medium containing 10% FBS were added to each pre-coated well. For each mice, 200 ng of H5 or GFP antigen and 100 anti-mouse CD28 (BD Pharmingen) were added to triplicate wells as sample stimulatory agents; 2 ng phorbol 12-myristate 13-acetate (PMA, Sigma), 100 ng Ionomycin calcium salt (INO, Sigma) and 100 ng anti-mouse CD28 were added into triplicate wells as the stimulatory agents of positive control; Meanwhile, another triplicate wells was added no stimulatory agents as negative control. Plates were then incubated at 37°C for 44 hours. Wells were then washed 5 times with 200 μl PBS, and incubated with 100 μl of 1 μg/ml detection antibody (R4-6A2-biotin, Mabtech) in PBS-0.5% FBS for 2 hours at room temperature. Unbound antibody was removed by washing 5 times with 200 μl PBS, then plates were incubated with 100 μl of 1000-fold diluted Streptavidin-HRP in PBS + 0.5% FBS for 1 hour at room temperature. After washing 5 times with 200 μl PBS, 100 μl of TMB solution was added, and plates were allowed to develop at room temperature until distinct spots emerged. Colour development was stopped by washing extensively with water. After allowing spots to dry at room temperature in a fume hood, plates were analyzed using an ELISpot reader (C.T.L. Analyzers, LLC).

#### Statistical analysis

Differences between the means of two experimental groups were analyzed by the Student’s *t*-test. One-Way ANOVA (SPSS 16.0, SPSS Inc., Chicago, USA) was performed to compare the differences of multiple groups. Results are presented as mean values ± standard error, and at least three independent replicates were performed for each condition.

## Abbreviations

EGFP: Enhanced green fluorescent protein; HA: A fragment of the hemagglutinin protein from the H5N1 influenza virus; HAOP: A synthetic codon-optimized HA epitope gene; IFN-*γ*: Interferon-gamma; CFU: Colony forming unit; ELISA: Enzyme-linked immunosorbent assays; HIA: Hemagglutination Inhibition Assays; APCs: Antigen-presenting cells.

## Competing interests

They authors declare that they have no competing interests.

## Authors’ contributions

SYZ, BY, JDH, KYY and BJZ designed the experiments. SYZ, BY, KZ, MC, YHH and SFY performed the experiments. SYZ, BY and JDH analyzed the data. SYZ, BY, RMW, KYY, BJZ and JDH drafted and revised the manuscript. SYZ and BY contribute equally to this manuscript. All the authors read and approved the final manuscript.
